# Epigenetics and Vascular Diseases: Influence of Non-coding RNAs and Their Clinical Implications

**DOI:** 10.3389/fcvm.2017.00026

**Published:** 2017-04-27

**Authors:** Leonardo Elia, Manuela Quintavalle

**Affiliations:** ^1^Humanitas Clinical and Research Center, Milan, Italy; ^2^Department of Molecular and Translational Medicine, University of Brescia, Brescia, Italy

**Keywords:** microRNAs, long-non-coding RNAs, vascular diseases, RNA therapeutics, gene expression

## Abstract

Epigenetics refers to heritable mechanisms able to modulate gene expression that do not involve alteration of the genomic DNA sequence. Classically, mechanisms such as DNA methylation and histone modifications were part of this classification. Today, this field of study has been expanded and includes also the large class of non-coding RNAs (ncRNAs). Indeed, with the extraordinary possibilities introduced by the next-generation sequencing approaches, our knowledge of the mammalian transcriptome has greatly improved. Today, we have identifying thousands of ncRNAs, and unsurprisingly, a direct association between ncRNA dysregulation and development of cardiovascular pathologies has been identified. This class of gene modulators is further divided into short-ncRNAs and long-non-coding RNAs (lncRNAs). Among the short-ncRNA sub-group, the best-characterized players are represented by highly conserved RNAs named microRNAs (miRNAs). miRNAs principally inhibit gene expression, and their involvement in cardiovascular diseases has been largely studied. On the other hand, due to the different roles played by lncRNAs, their involvement in cardiovascular pathology development is still limited, and further studies are needed. For instance, in order to define their roles in the cellular processes associated with the development of diseases, we need to better characterize the details of their mechanisms of action; only then might we be able to develop innovative therapeutic strategies. In this review, we would like to give an overview of the current knowledge on the function of ncRNAs and their involvement in the development of vascular diseases.

## Introduction

**Figure 1:**
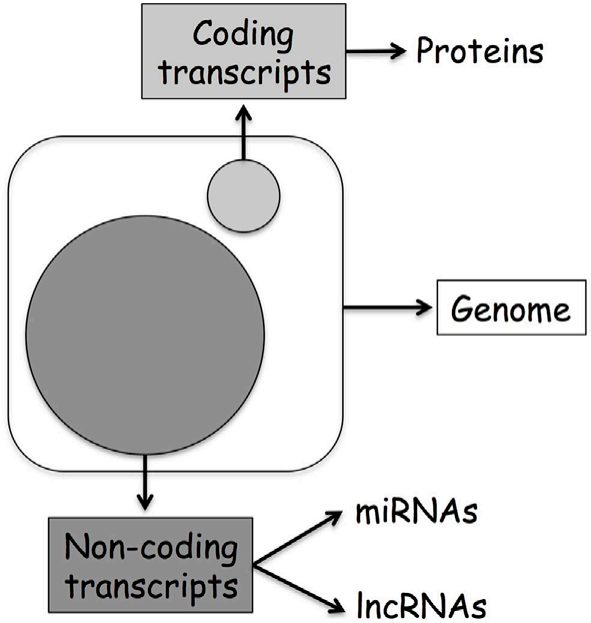
**Human genome organization: coding vs. non-coding transcripts**. The large circle represents ncDNA, while the small coding DNA.

Originally, biologists assumed that the direction of information within a cell was a linear process going from DNA to RNA to protein and named this concept as the central dogma of molecular biology (1). However, in the last decades, the discovery of additional mechanisms able to modulate DNA transcription has clearly demonstrated that the layers of gene regulation are more complex. Modulation of gene expression might happen by direct methylation of DNA or alteration of DNA accessibility through histone modifications, events refer to as classical epigenetics. On the other hand, recent discoveries originating from the human genome project first, and then from the data generated through the next-generation sequencing approach, suggest that even more layers of regulation characterize this epigenetic landscape. Today, we know that only 2% of our DNA is translated into protein, and that the previously defined “junk RNA” is instead a fundamental part of the machinery involved in gene regulation (Figure 1). Thereby, it is clear that a new genomic era has started, and we can name this as the “non-coding RNA (ncRNA) revolution.” This provides an important new perspective on the centrality of RNA in gene regulation.

Non-coding RNAs can be divided into two main classes: small-ncRNAs (<200 nucleotides long), which includes microRNAs (miRNAs), piwi-interacting RNAs, and short-interfering RNAs; and long-non-coding RNAs (lncRNAs) (>200 nucleotides long), which include natural antisense transcripts, small nucleolar RNAs, and other types of lncRNAs (2–5) (Table 1).

**Table 1:** **Classes of non-coding RNAs**.

Non-coding RNA	Symbol	Functions
**Short-ncRNAs**		
microRNA	miRNA	Posttranscriptional regulators
Piwi-interacting RNA	piRNA	DNA methylation, transposon repression
Short-interfering RNA	siRNA	RNA interference
**Long-non-coding RNAs**		
Long-intervening ncRNA	lincRNA	Epigenetic regulators of transcription
Small nucleolar RNA	snoRNA	Nucleotide modification
Circular RNA	circRNA	miRNA sponging and RNA polymerase II regulators

Even though the biology of ncRNAs is not completely understood, the time to generate therapeutics based on RNA biology is fast approaching. Indeed, recent discoveries on the role of ncRNAs have completely changed our view of cell biology. Until few decades ago, we thought that phenotypes depended only on genetic variants, but the existence of non-Mendelian inheritance indicates that this view is not completely correct. In addition to this, the improvements in RNA stabilization chemistry and the ability to make informed decisions about where a defined molecule should perform its action is allowing progress in the generation of these new types of drugs. In this review, we focus in particular on the role of miRNAs and lncRNAs in vascular biology and disease.

## microRNAs

microRNAs are a class of endogenous ncRNA between 16 and 29 nucleotides long. miRNAs are very well conserved through different species, but they do not always regulate the same genes in different organisms. Generally, miRNAs negatively regulate gene expression by binding to the 3′UTR of their target mRNAs, inhibiting transcription or translation (6). Even if rare, they might also block gene expression using alternative methods to 3′UTR binding, such as deadenylation of the polyA tail of the targeted mRNA (7). Furthermore, few studies demonstrated also that miRNAs might bind the promoter of some genes, enhancing RNA polymerase II (PolII) recruitment and thereby increasing gene transcription (8–10).

microRNAs are regarded as central players in physiology and pathophysiology of the vascular system (11–13). For different reasons, they are interesting targets for therapeutic intervention. A single miRNA can modulate hundreds of genes, including targets in the same pathway; thereby the modulation of their activity might have a major effect on cellular processes. miRNAs are essential regulators of vascular functions, playing a central role in endothelial cell (EC) and smooth muscle cell (SMC) biology (12–14). Finally, the idea to generate RNA therapeutics benefits from previous experience in the field of RNAi (15), that even though includes some specific limitations (16), might hugely impact the generation of a new class of drugs.

Vascular homeostasis is fundamental for the efficiency of the entire cardiovascular system. Pathological alteration of vascular ECs and SMCs leads to the development of different diseases, including atherosclerosis, restenosis, and even diabetes (17, 18). A dysfunctional endothelium contributes to increased stiffness of vessels and impaired distensibility of the arteries and also alters the amount of energy and oxygen transport to cardiac cells, consequentially triggering myocardial damage (19). It has been demonstrated that alteration of EC miRNA biogenesis by knocking down Dicer, a key enzyme involved in miRNA maturation, induces endothelial dysfunction (20, 21). miRNA-126, miRNA-92, and recently the miRNA-143/145 cluster have been shown to play a critical role on EC biology. Through zebrafish and mouse loss-of-function models, miRNA-126 has been shown to regulate the angiogenic ability of ECs. In both models, altered vessel organization during embryogenesis and adulthood was observed (22, 23). A negative effect on vessel angiogenesis is instead played by miRNA-92a: in a mouse model of myocardial infarction, forced modulation of its expression by antagomiRs accelerated vessel growth, and then recovery of cardiac functionality (24). Instead, miRNAs not usually expressed by ECs might be activated by alteration of laminar blood flow, such as the so-called SMC-specific miR-143/145 cluster. miRNA activation is regulated at the transcriptional level, by Kruppel-like factor 2 (KLF2) (25), and posttranscriptionally, by modulation of AMP-activated protein kinase α2, leading to EC-dependent vascular complications (26).

Alteration of the SMC miRNome might lead to vascular complications as well. For instance, neointimal formation, mainly due to SMC dysregulation, is a hallmark of many vascular diseases, including atherosclerosis (27) and pulmonary hypertension (28). The balloon injury model is a common-used model for the study of vascular restenosis, and many miRNAs have been identified by this approach. miRNA-21 has been shown to promote SMC proliferation and migration, inhibiting apoptotic pathways regulated by tropomyosin 1 (29), PTEN, and Bcl-2 (30). Transforming growth factor beta (TGFβ) is a central mediator of vascular remodeling processes (31) following acute injuries; miR-21 has been demonstrated to be regulated by TGFβ at the posttranscriptional level (32), highlighting the potential to generate RNA therapeutics vs. this miRNA. Indeed, through genetic ablation of miR-21 in the mouse, it was possible to reduce neointimal formation in vein graft failure (33) and in-stent restenosis models (34). Similar results were obtained using anti-miRNA technologies (35) (the alternative chemistries to generate therapeutics will be discussed later).

Among the list of the most expressed and fundamental miRNAs for SMCs is also the miRNA-143/145 cluster. It was originally associated with cancer development (36, 37), then more recently it has been demonstrated to play a central role on SMC biology, regulating SMC fate during development (38) and SMC phenotypic switch in adulthood (39). These features of the cluster have been demonstrated by different approaches, including gene therapy (40), but also through the generation of a knock-out mouse model (41–45), in which reduced neointimal formation was observed. Similar results have been obtained also by modulating miRNA-133 expression. Even though its expression in SMCs is not comparable to that of the miRNA-143/145 cluster, it is able to regulate SMC transition from the contractile to the proliferative phenotype. Indeed, its over-expression in rat carotid artery using adenoviral vectors reduced balloon injury-induced neointimal formation (46).

Cell-to-cell communication can be mediated by miRNAs transported by different carriers. Recent reports have shown the involvement of proteins, extracellular vesicles, and membrane structures in miRNA-mediated cell-to-cell cross talk. miRNAs, circulating in the blood stream protected by high-density lipoprotein, can be delivered to recipient cells where they inhibit target mRNAs (47); in addition, in plasma, miRNAs can form complexes with Argonaute 2 (48). Extracellular vesicles seem also to be involved in miRNA transport, and known players identified in this category are apoptotic bodies (diameter raging between 800 and 5,000 nm) (49) and exosomes (diameter < 150 nm) (50). Indeed, Zernecke and colleagues demonstrated that EC-derived apoptotic bodies are able to vehicle miRNA-126 to other cells in order to protect injured vessels from atherosclerosis development (51). Similarly, miRNA-126 might be transferred from ECs to SMCs also *via* exosomes to regulate cell turnover of the recipient cells (52). Actually, the importance of exosomes in cell-to-cell miRNA-mediated communication has been highlighted by many other reports. For instance, a recent work of Dimmeler’s group showed the involvement of these small vesicles in the transfer of the miRNA-143/145 cluster from ECs to SMCs (25). KLF2 induces the secretion of miRNA-143/145-enriched exosomes that are then taken up by SMCs, inducing an atheroprotective SMC phenotype through inhibition of specific target genes. In the opposite direction, miRNA-143 might, by transfer from SMCs to ECs of the pulmonary vasculature, induce a pro-migratory, angiogenic EC phenotype (53). Apart from exosome-mediated transfer, we recently showed that TGFβ triggers miRNA-143/145 cluster transfer from SMCs to ECs. We showed that its transfer is mediated by tiny cytoplasmic structures known as tunneling nanotubes. Once in the receiving cells, the miRNA-143/145 cluster modulates hexokinase 2 and integrin beta-8, negatively modulating EC proliferation and angiogenesis, thereby influencing vessel stabilization (54).

In summary, different miRNAs have been shown to be involved in vascular remodeling processes *in vitro* and *in vivo*. There are a lot of opportunities to develop therapeutics, but many facts of miRNA biology need to be elucidated in order to reduce potential long-term side effects.

## Long-non-coding RNAs

Accumulating evidence suggests that lncRNAs play an important role in development and diseases through epigenetic control over gene expression (55, 56). LncRNAs are usually transcribed by PolII, spliced, 5′-capped, and 3′-polyadenylated, similarly to protein-coding mRNAs (57). The low level of lncRNA sequence homology between human and other species has been the subject of a long debate, but now conservation is usually evaluated in terms of secondary structure, highlighting a higher conservation level between species (58). Originally, it was thought that lncRNAs did not have any coding potential, while recent pioneer studies have demonstrated that they might actually encode for small peptides (59–61).

**Figure 2:**
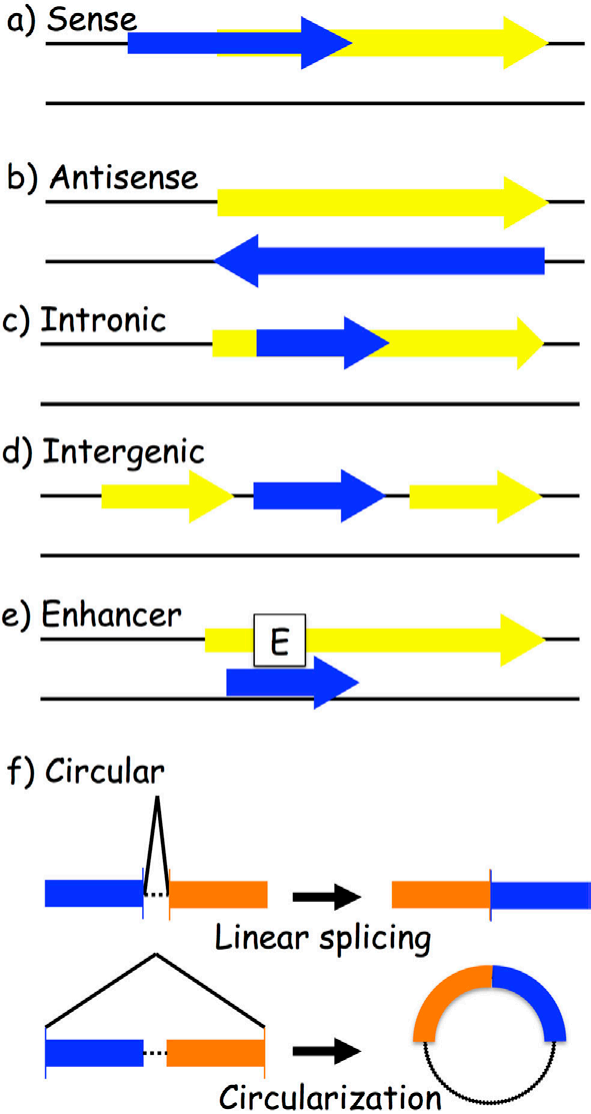
**Biogenesis of long-non-coding RNAs (lncRNAs)**. LncRNA transcripts (blue box) are classified based on their genomic location in relation to the closest gene (yellow box): (a) sense lncRNAs are transcribed on the same strand as an exon; (b) antisense lncRNAs are transcribed on the opposite strand of an exon; (c) intronic lncRNAs; (d) intergenic lncRNAs are located between two different genes; (e) enhancer lncRNAs arise from an enhancer region of a protein-coding gene; and (f) circular RNAs are exonic or intronic sequences that circularize following an alternative splicing of linear transcripts.

One way to classify lncRNAs is through their association with nearby protein-coding genes. The following are the most accepted categories of ncRNAs: (a) sense and (b) antisense, transcribed on the same or opposite strand of a protein-coding gene; (c) intronic and (d) intergenic, arising from an intron of a protein-coding gene or from a region located between two protein-coding genes; (e) enhancer, located in the enhancer region of a protein-coding gene; and (f) circular RNAs (circRNAs), a covalently closed RNA usually deriving from an alternative splicing of a protein-coding gene (Figure 2). LncRNAs play a wide range of roles in the cell, such as transcriptional and posttranscriptional regulation of gene expression. Based on their location, we distinguish between nuclear and cytoplasmic lncRNAs. In the nucleus, they might act as modifiers of chromatin, being involved in the spatial localization of DNA-associated proteins to specific genomic loci. For instance, they might show an enhancer-like activity, inducing transcription (62), or do the exact contrary, inhibiting gene expression by recruiting DNA and histone methyltransferases (63) or histone modifiers, such as the polycomb repressive complex 2 (64) and histone H3 lysine 9 methyltransferases (65). Cytoplasmic lncRNAs might work as activators or repressors of gene expression as well. Positive gene regulation might be obtained when working as miRNA sponges: specific miRNA-target genes are then de-repressed (66–68). On the contrary, protein inhibition might take place by binding to complementary mRNA sequences (69, 70).

In the last decade, our understanding on the roles of lncRNAs in pathologies such as cancer has grown exponentially, whereas knowledge on their role in vascular diseases remains limited.

Interaction between SMCs and ECs is critical for vessel physiology (71), thereby the identification of lncRNAs playing a role on both cell types is of great interest. For instance, the vascular-enriched lncRNA SENCR has been shown to regulate the differentiation status of SMCs, downregulating myocardin, and several contractile proteins. Moreover, SMCs with a decreased expression of SENCR acquire a pro-migratory phenotype, through the upregulation of midkine and pleiotrophin (72). On the other hand, the same lncRNA regulates proliferative, migratory, and angiogenic capabilities of ECs (73). Importantly, the same studies showed that expression of SENCR is altered in samples from patients with vascular diseases, including limb ischemia and coronary disease.

Evidence shows that lncRNA-p21 is downregulated in the coronary vessels of patients with heart disease (74). *In vitro* studies confirmed the ability of lncRNA-p21 to inhibit SMC proliferation and to induce cellular apoptosis. Similarly, lncRNA-p21 reduces EC proliferation and increases cellular apoptosis in *in vitro* models, mainly through the regulation of miRNA-130 (75). Moreover, Wu and colleagues have downregulated lncRNA-p21 *in vivo*, observing increased neointimal hyperplasia formation in injured carotids (74).

Smooth muscle cell-enriched or EC-enriched lncRNAs have been identified as well. Recently, Baker’s group, through RNA-sequencing analysis, identified an SMC-specific lncRNA named SMILR, which regulates SMC proliferation. The authors showed the clinical association between the level of SMILR in plasma and the stability of atherosclerotic plaques (76). Through a similar approach (RNA sequencing), several lncRNAs modulated by angiotensin II have been identified in SMCs. In particular, lnc-Ang362, a host transcript for miRNA-221 and miRNA-222, regulates the expression of these two miRNAs, resulting in a decrease of SMC proliferation (77).

Hypoxia regulates the metastasis-associated lung adenocarcinoma transcript 1 (MALAT1) in ECs. Inhibition of MALAT1 *in vitro* activates migration of EC tip cells, while proliferation of EC stalk cells is disturbed, resulting in impaired angiogenesis, as observed also in retina vascularization and collateral vessel formation in ischemic limbs (78).

Very recently, a new class of lncRNAs named circRNAs has been identified. They can be either exonic or intronic in origin. CircRNAs derive from alternative splicing of linear genes, and they might play different functions in relation of their origin (79). Exonic circRNAs are predominately located in cytoplasm, and they have been shown to bind miRNAs, modulating their direct effects on specific target genes (80). For instance, circRNA-7 contains more than 70 binding sites for miRNA-7, acting as its natural cellular sponge (67, 68). On the other hand, intronic circRNAs are mainly located in the nucleus and are poorly enriched in miRNA-binding sites (79). A recent report by Zhang and colleagues showed an ability of circ-ankrd52 to regulate its parental gene by stabilizing the PolII machinery (81). Moreover, other reports showed the enrichment of circRNAs in exosomes; their detection the plasma of patients suggests a potential role as clinical biomarkers (82, 83).

However, as of today, only one study has been published on the role of circRNAs in vascular cells, with particular attention to regulation by hypoxia in ECs (84). This calls for a more detailed analysis of their role in vascular development and disease.

## Therapeutic Prospective for ncRNAs

The need to translate ncRNA findings from bench to bedside is a direct consequence of the need to identify new therapeutic approaches to treat vascular diseases. For this purpose, different technologies are currently available. In particular, miRNA therapeutics have already entered phase I clinical studies.

*In vivo* modulation of miRNA expression might be reached in different ways. For instance, lentiviral or adenoviral vectors carrying specific miRNAs have been utilized in animal models of vascular diseases. Over-expression of miRNA-145 by this approach reduced neointimal formation and atherosclerosis development in rodents (40, 42, 85). However, even if this approach might be optimized for specific local delivery using stent technologies or similar, concerns have been raised on the use of this method in humans. Thereby, the most developed methodology for miRNA modulation is based on chemically modified oligonucleotides. In order to increase miRNA levels, mimic oligonucleotides might be used. In a model of pulmonary hypertension in mice, Montgomery and colleagues systemically delivered miRNA-29 conjugated with cholesterol, observing a reduction of pulmonary fibrosis (86).

On the other hand, normalization of an abnormally upregulated miRNA might be obtained utilizing antisense molecules. Different chemistries are available for this purpose: the first used technology was named “antagomiR,” in which the modified miRNA antisense oligo is conjugated to 2′-*O*-methyl-cholesterol, while the entire molecule is stabilized by phosphorothioate bonds (87). More recently, pharmaceutical companies have started to produce different oligonucleotides to improve miRNA targeting. For instance, Santaris patented a chemistry called “locked nucleic acids” (LNAs) in which the oligonucleotides are shorter than the target miRNA (8- or 15-base antisense molecules), thereby making them easier to deliver and without the need for cholesterol conjugation. Similarly, lncRNAs might be inhibited using molecules names GapmeRs: these are antisense oligonucleotides in which DNA monomers are flanked by LNA-modified sequences. The LNA stretches increase the stability of the oligonucleotide, while DNA gaps trigger the activity of RNase H due to the presence of DNA:RNA duplexes (88).

In term of clinical applications, there are only three ongoing clinical trials. The most advanced is a phase IIa clinical trial on anti-miRNA-122 by Santaris (http://ClinicalTrials.gov identifier: NCT01200420): the anti-miRNA-122 LNA inhibits hepatitis C replication through miRNA-122 reduction in liver (89–91). Mirna Therapeutics is instead running a phase I study in which miRNA-34a is delivered through liposomal injections to treat solid cancers in patients with liver involvement or inoperable primary liver cancer (http://ClinicalTrials.gov identifier: NCT01829971) (92). Finally, Regulus Therapeutics very recently started a phase I clinical study on an anti-miRNA-21 molecule to treat Alport syndrome, a kidney disease that alters the glomerular basement membrane (http://ClinicalTrials.gov identifier: NCT02855268) (93).

Several preclinical studies have confirmed the potentiality to develop miRNA-based therapeutics, and this has been confirmed by the above-described clinical trials. However, to take full advantage of this approach, many questions remain open: for example, what is the role of the miRNA passenger strand, since it has been demonstrated that in some cases it has a biological function. On the other hand, our knowledge on lncRNAs is still limited, in particular related to vascular diseases. Consequently, clinical application of lncRNA modifiers is much more distant and more studies are needed.

## Author Contributions

LE and MQ equally contributed to the preparation of the submitted mini review.

## Conflict of Interest Statement

The authors declare that the research was conducted in the absence of any commercial or financial relationships that could be construed as a potential conflict of interest. The reviewer, CP, and handling Editor declared their shared affiliation and the handling Editor states that the process nevertheless met the standards of a fair and objective review.
